# Assessing Functional Disability in Glaucoma: The Relative Importance of Central Versus Far Peripheral Visual Fields

**DOI:** 10.1167/iovs.61.13.23

**Published:** 2020-11-17

**Authors:** Jamie L. Odden, Aleksandra Mihailovic, Michael V. Boland, David S. Friedman, Sheila K. West, Pradeep Y. Ramulu

**Affiliations:** 1 Eye and Ear Institute, University of Pittsburgh Medical Center, Pittsburgh, Pennsylvania, United States; 2 Wilmer Eye Institute, Johns Hopkins University, Baltimore, Maryland, United States; 3 Dana Center for Preventive Ophthalmology, Johns Hopkins University, Baltimore, Maryland, United States

**Keywords:** disability, function, visual field, far periphery, automated perimetry

## Abstract

**Purpose:**

To evaluate the importance of central versus far peripheral visual field (VF) loss in assessing disability in glaucoma.

**Methods:**

In total, 231 patients with glaucoma or suspected glaucoma completed 24-2 VF testing and automated peripheral VFs using the suprathreshold 30- to 60-degree pattern. Questionnaires assessed fear of falling (FoF), quality of life (QOL), instrumental activities of daily living (IADLs), and driving habits; nonsedentary time, reading speed, and gait were objectively measured. Multivariable regression models analyzed the effect of central VF and/or peripheral VF damage on each outcome.

**Results:**

In models including both central and peripheral VF damage (independent effects), greater central, but not peripheral, VF damage was associated with greater FoF, worse QOL, fewer daily steps, and difficulty with IADLs (*P* < 0.02 for central; *P* > 0.5 for peripheral). For gait measures, greater peripheral, but not central, damage was associated with shorter steps and shorter strides, as well as greater variability in step length (*P* < 0.03 for peripheral; *P* > 0.14 for central). Model *R*^2^ values were not substantially higher (less than 5% additional explained variability) for models including both central and peripheral VF damage as compared to the best models incorporating only one region of VF damage (i.e., central or peripheral).

**Conclusions:**

The relative importance of central 24 degrees versus more peripheral VF damage differs across functional domains in patients with glaucoma. Central damage is more strongly associated with most disability outcomes, although peripheral damage is more associated with specific gait measures. Studies examining the relative importance of various VF regions should assess functional domain separately and eschew integrated measures of quality of life/activity limitation.

Assessing functional disability associated with glaucoma can give clinicians crucial insight into the likely level of difficulty with important tasks that a patient faces daily and also can guide the appropriate level of risk for a patient's treatment given their stage of disease and degree of impairment.^1^ Visual field (VF) testing is frequently used to assess the functional impact of glaucoma, and studies relating VF damage to disability have predominantly used central VF tests that are used in clinical practice, although glaucomatous damage sometimes begins more peripherally to this tested region.

VF loss outside the central 24 degrees, although more difficult to measure due to greater variability in normal sensitivity with greater eccentricity from fixation, may predict specific functional outcomes that rely more on one's peripheral vision than central vision.^2^^,^^3^ Freeman et al.^4^ reported that peripheral VF loss outside the central 30 degrees was associated with greater odds of falling in a population-based sample of older adults, while VF loss within 30 degrees of fixation was not, and it is reasonable that VF loss in the far periphery may best predict visual disabilities that require hazard detection (i.e., driving, mobility, and judging the safety to cross a street).^5^ However, a paucity of literature has evaluated both central and peripheral vision as predictors of disability, with most studies focusing on more central visual field damage (10-2 vs. 24-2), ignoring the far periphery.^6^^,^^7^ Here, we examine the effect of central and more peripheral VF damage on several individual disability measures such as driving and physical activity level. Our approach contrasts with prior studies that have examined quality of life (QOL) as a single construct in a multi-item questionnaire,^8^^–^^10^ and approach subject to error as it ignores the relative importance of VF damage in different regions on different aspects of QOL.

Here, we sought to evaluate the extent to which central and far peripheral VF damage assess functional disability in patients with a wide spectrum of glaucoma severity. We evaluated VF damage in the central 24 degrees and far periphery beyond 30 degrees using static automated testing. A variety of functional measures were analyzed, including fear of falling (FoF), gait, quality of life, driving habits, maximum reading speed, and physical activity level. We hypothesized that more VF damage in the far periphery would better capture disability in tasks that are more dependent on peripheral vision, such as driving, gait, and physical activity, while VF damage in the central region would be more strongly associated with tasks of central vision such as reading speed.

## Methods

The study was approved by the Johns Hopkins University School of Medicine Institutional Review Board and adhered to the Declaration of Helsinki. Written informed consent was obtained from all participants.

### Study Design, Setting, and Population

Patients were enrolled as a part of the Falls in Glaucoma study from September 2013 through March 2015 at the glaucoma clinic of the Johns Hopkins Wilmer Eye Institute. Participants were included if they were 60 years or older sometime during the 3-year study and had a diagnosis of primary open-angle, primary angle closure, pseudoexfoliative, or pigmentary glaucoma. Patients with suspected glaucoma based on the presence of intraocular pressure elevation, family history, narrow angles, pseudoexfoliative material, or pigment dispersion were also included. Patients with concurrent eye diseases (i.e., age-related macular degeneration, uveitis, retinal detachment) resulting in visual acuity worse than 20/40 in either eye were excluded, as were those with neurologic disorders, prior pan-retinal photocoagulation, or other disorders resulting in VF damage. Additional exclusion criteria included (1) any laser eye treatment or surgery (ocular or nonocular) in the past 2 months, (2) any hospitalization in the past month, (3) bed or wheelchair confinement, and (4) inability to complete VF testing.

### Central and Peripheral VF Testing

Participants performed two types of VF testing on a single Humphrey Field Analyzer IIi (Carl Zeiss Meditec, Inc., Dublin, CA, USA): (1) the 24-2 Swedish interactive thresholding algorithm (SITA) standard test (reflecting central testing) and (2) the suprathreshold peripheral 60 screening test pattern (reflecting peripheral testing) with 60 test points displayed between 30 and 60 degrees of eccentricity from fixation. Tests were done on different days, with the central test slightly preceding the peripheral test (median, 2.0 months; interquartile range, 0.6–6.7 months).

For the peripheral 60 screening test pattern, eyes were presented a stimulus 6 dB brighter than the expected sensitivity at given test location points, and stimuli were recorded as seen or unseen. Further details of central and peripheral VF testing have been previously described.^2^^,^^11^

### Converting Central and Peripheral Tests to a Similar Scale and Integrating VFs

Central and peripheral VFs of both eyes were combined to create integrated VFs for each test location. Central test points were first dichotomized as being normal or abnormal, with a pattern deviation value worse than 6 dB defined as abnormal.^2^ To eliminate underestimation of damage in eyes with diffuse loss pattern deviation, values were recalculated for 10% of central VFs to allow a maximum of 5 dB added to total deviation values when deriving pattern deviation values. Peripheral VF testing already accounts for the possibility of diffuse, severe loss by limiting the degree to which the expected sensitivity is shifted (by modeling the hill of vision), so pattern deviation values were used (instead of total deviation values), and no further adjustments were necessary. For both tests, VF points in the left and right eye were classified as abnormal versus normal. For each spatial (x-y) coordinate, if both right and left eye points corresponding to this coordinate were abnormal, then that coordinate was classified as abnormal; otherwise, the coordinate was considered normal.^12^

Some patients only underwent VF testing in one eye due to severe vision loss in the contralateral eye, in which case the value of each integrated visual field (IVF) point was derived solely from the tested eye (20 eyes; 11 for central and 9 for peripheral testing). An eye was excluded if both central and peripheral testing were not performed on it (9 eyes lacked peripheral testing; 9 eyes lack central testing, resulting in *n* = 231).

### Functional Outcomes Assessment

Baseline data regarding the following outcomes were collected: fear of falling, quality of life, daily steps, nonsedentary time, reading speed, driving cessation, driving limitations, difficulty with instrumental activities of daily living (IADLs), and gait characteristics (base of support, step length, stride length, stride velocity, walking speed, variability in step length, variability in stride length, and variability in stride velocity).^12^

Fear of falling was evaluated by asking participants how worried they would be about falling if they were to perform 18 different tasks.^13^ Quality of life was evaluated with the Glaucoma Quality-of-Life 15 scale. Using Winsteps, version 3.93.2 (Winsteps, Chicago, IL, USA). Rasch analysis was performed for both measures to estimate person measure scores expressed in a log-odds units (logits). Higher person measure scores reflect participants with lower ability (greater fear of falling or more task difficulty).

Participants wore accelerometer devices (Actical; Respironics Inc., Murrysville, PA, USA) during a typical week to measure physical activity. Daily steps were calculated by dividing the total trial steps by the number of days the device was worn. Nonsedentary time was defined as the sum of the average number of minutes spent in low, moderate, or vigorous physical activity per day, using previously described activity level thresholds.^14^^,^^15^

Reading speed was assessed by measuring aloud using the MNRead chart,^16^ with maximum reading speed calculated in words per minute (wpm) using nonlinear mixed-effects models.^17^

Gait characteristics were assessed by having participants walk barefoot at their natural pace along the GAITRite Electronic Walkway (CIR System, Inc., Franklin, NJ, USA), as previously described.^12^^,^^18^

Driving habits (driving cessation and driving limitations) were assessed by questionnaire.^19^ A patient was considered to have ceased driving if they had not driven a car in the past year. Nine driving limitations were queried and summed to create a limitations score.^17^

IADLs were assessed using previously described questionnaires and methods.^20^ Participants were considered to have a disability for a specific IADL if they reported performing the task with help or were unable to perform it at all. IADLs were reclassified as a binary variable: no IADL difficulty versus any difficulty in one or more IADLs.

### Evaluation of Covariates

Age, gender, race, living status (living alone versus with another), employment status, education level, depression, and mini-mental status were gathered using standard questionnaires. Non–eye drop medication lists were generated by direct observation of pill containers when possible, or otherwise by patient report, and classified as polypharmacy if five or more non–eye drop prescription medications were used.^21^ Patients were questioned about 15 comorbid medical conditions known to affect physical activity using a standardized questionnaire, and comorbidity was quantified as the total number of comorbid conditions, as previously described.^14^ The small number of participants with more than five comorbidities (*n* = 9) were reclassified to have five comorbidities. Height and weight were collected at baseline to calculate participants’ body mass index (BMI). Grip strength was measured by having participants squeeze the Jamar Hand Dynamometer (Sammons Preston Rolyan, Bolingbrook, IL, USA) as hard as possible with their dominant hand. Leg strength was measured by having participants flex the hip to push their thigh against the MircoFET2 Dynamometer (Hoggan Scientific LLC, West Jordan, UT, USA) while sitting in a chair. Strength for both grip and leg strength was recorded in kilograms of force.

### Statistical Analysis

Multivariable regression models were created separately for each disability outcome. Regression model was linear for fear of falling, quality-of-life scores, maximum reading speed, and all gait measures; negative binomial for daily steps and nonsedentary time; logistic for IADL difficulty and driving cessation; and ordinal logistic for driving limitations.

Age, gender, race, and comorbidities were included as covariates in all regression models. Models included additional covariates as follows: (1) fear of falling: living status, BMI, grip strength, depression, and leg strength; (2) quality of life: depression; (3) daily steps and nonsedentary time: education level, depression, BMI, and mini-mental status; (4) reading speed: education level, depression, and mini-mental status; (5) driving cessation, driving limitations, and IADL difficulty: education level, mini-mental status, depression, and grip strength; and (6) all gait measures: polypharmacy.

To assess the level of collinearity between central and peripheral IVFs, we calculated variance inflation factors (VIFs) for each regression model. VIFs less than 4 reflect acceptable collinearity,^22^ and VIFs for central and peripheral IVF sensitivity demonstrated values below this threshold for all models, with a maximum value of 2.62.

Data were analyzed using STATA version 13 (StataCorp LP, College Station, TX, USA).

## Results

Most of the 231 participants were between ages 64 and 75 years and Caucasian (71%) with a median better-eye mean deviation of –2.51 dB and a median worse-eye mean deviation of –5.16 dB (Table 1). The mean percentage of abnormal central and peripheral IVF points was 1.9% and 5.4 %, respectively.

**Table 1.** Characteristics of 231 Study Patients

Characteristic	Value
Demographics	
Age, mean (IQR), y	70.6 (64, 75)
African American race	64 (28)
Male gender	117 (51)
Employed	84 (36)
Lives alone	46 (20)
Education	
Less than high school	7 (3)
High school	29 (13)
Some college	28 (12)
Bachelor's degree	56 (24)
More than bachelor's degree	110 (48)
Health	
Comorbid illnesses >1	151 (65)
Polypharmacy	75 (32.5)
Depressive symptoms	8 (3.5)
Body mass index, mean (SD), kg/m^2^	27.0 (5.0)
Vision	
MD better eye, median (IQR)	–2.51 (–4.41, –0.64)
MD worse eye, median (IQR)	–5.16 (–11.37, –2.62)
Abnormal central IVF points, median (IQR), %	1.92 (0, 13.46)
Abnormal peripheral IVF points, median (IQR), %	5.36 (1.79, 17.63)
Central IVF mean sensitivity, median (IQR), dB	27.90 (26.09, 29.67)
No. of abnormal peripheral IVF points, median (IQR)	11 (5, 19)

Values are presented as number (%) unless otherwise indicated. IQR, interquartile range; MD, mean deviation.

### Evaluation of Disability With Only Central or Peripheral Damage as an Independent Variable

**Figure 1.**
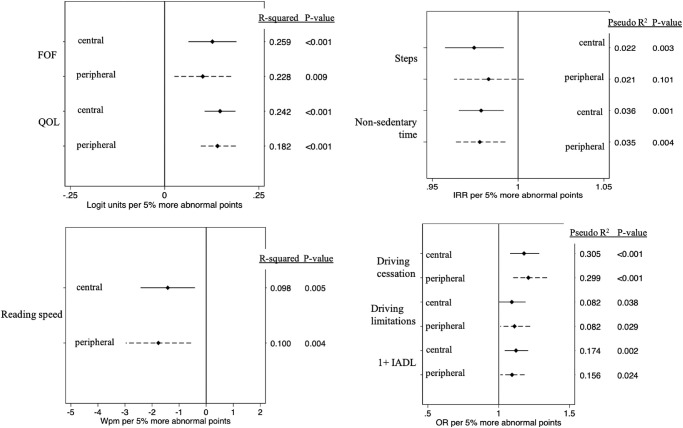
Isolated effects of 5% more abnormal central or peripheral points on each functional outcome.

Multivariable models with either central or peripheral percent abnormal points as an independent variable were first used to evaluate the effects of central or peripheral damage without considering damage outside of this region. Greater central and greater peripheral damage were both significantly associated with higher fear of falling levels, worse quality of life, and slower reading speeds (*P* < 0.01 for all); for the first two outcomes, model *R*^2^ values were higher (by 0.031 and 0.060) for models including central VF damage as compared to models including peripheral VF damage (Fig. 1 and Table 2). Greater central and greater peripheral damage were also both significantly associated with reduced nonsedentary time, a greater likelihood of driving cessation, a greater likelihood of difficulty with more than one independent activities of daily living, and more self-reported driving limitations (Fig. 1 and Table 2; *P* < 0.04 for all). Only central damage was associated with fewer daily steps (*P* < 0.01 for central; *P* > 0.1 for peripheral).

**Table 2.** Evaluation of Disability With Only Central or Peripheral Damage as an Independent Variable: Summary of Statistically Associated Functional Outcomes

Outcome Measure	Both	Central Only	Peripheral Only	Neither
FoF	X			
QOL	X			
Reading speed	X			
Steps		X		
Nonsedentary time	X			
Driving cessation	X			
Driving limitations	X			
1+ IADL	X			
Base of support			X	
Step length			X	
Stride length				X
Stride velocity				X
Walking speed				X
CV step length	X			
CV stride length	X			
CV stride velocity	X			

CV, coefficient of variation.

**Figure 2.**
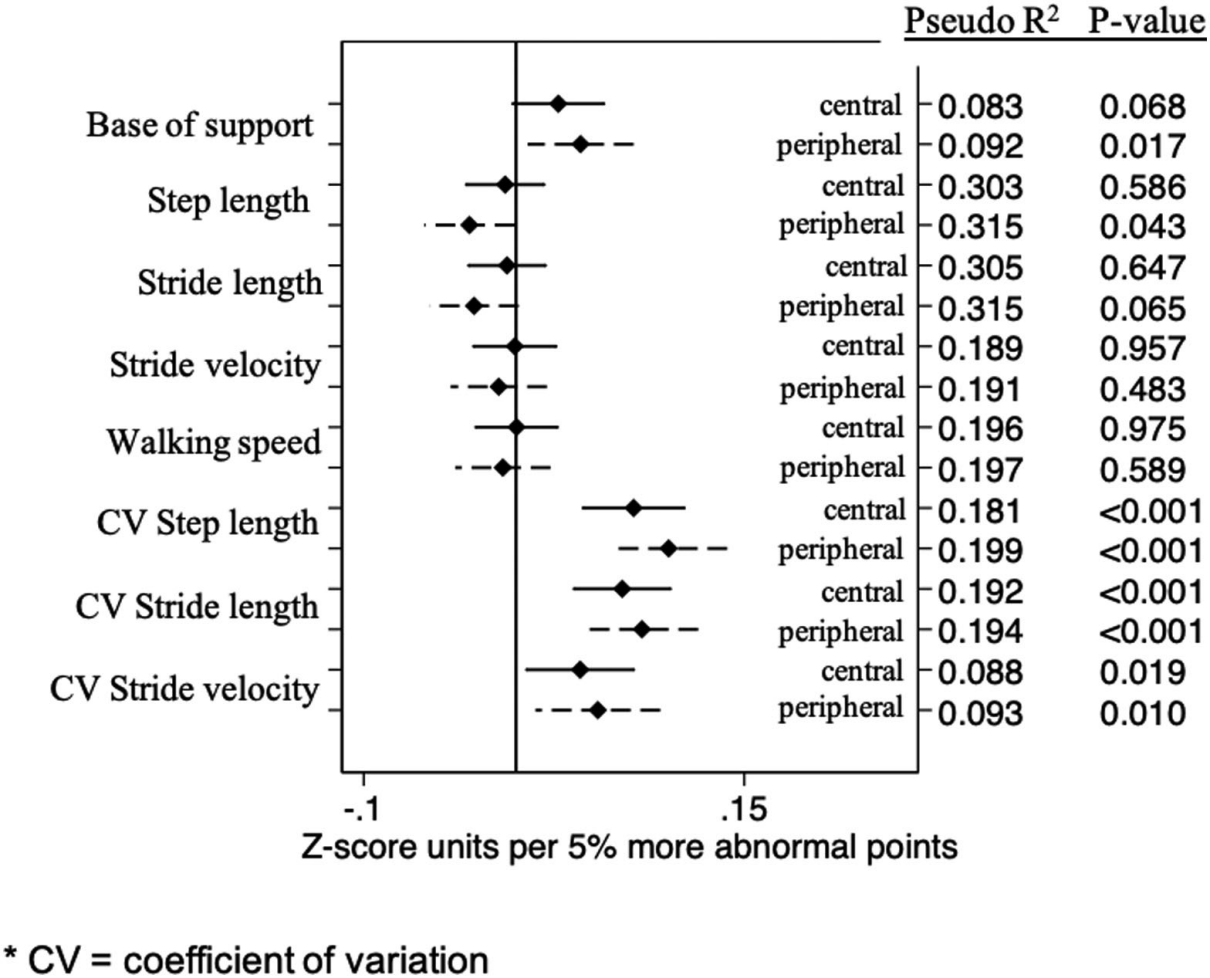
Isolated effects of 5% more abnormal central or peripheral points on each gait outcome.

With regards to gait, greater central and greater peripheral damage were both significantly associated with greater variability in step length, stride length, and stride velocity (*P* < 0.02 for all; Fig. 2 and Table 2). For each outcome, model *R*^2^ values were very similar for peripheral and central models (*R*^2^ ≤ 0.018 difference between models). Peripheral, but not central, damage was associated with shorter steps (Fig. 2 and Table 2; *P* = 0.04 for peripheral; *P* = 0.60 for central) and broader base of support (Fig. 2 and Table 2; *P* = 0.02 for peripheral; *P* = 0.07 for central). Neither central nor peripheral damage was associated with stride length, walking speed, or stride velocity (*P* > 0.06 for all). However, the overall differences in *P* values and *R*^2^ values were minor for each outcome.

### Evaluation of Disability With Central and Peripheral Damage as Independent Variables

**Figure 3.**
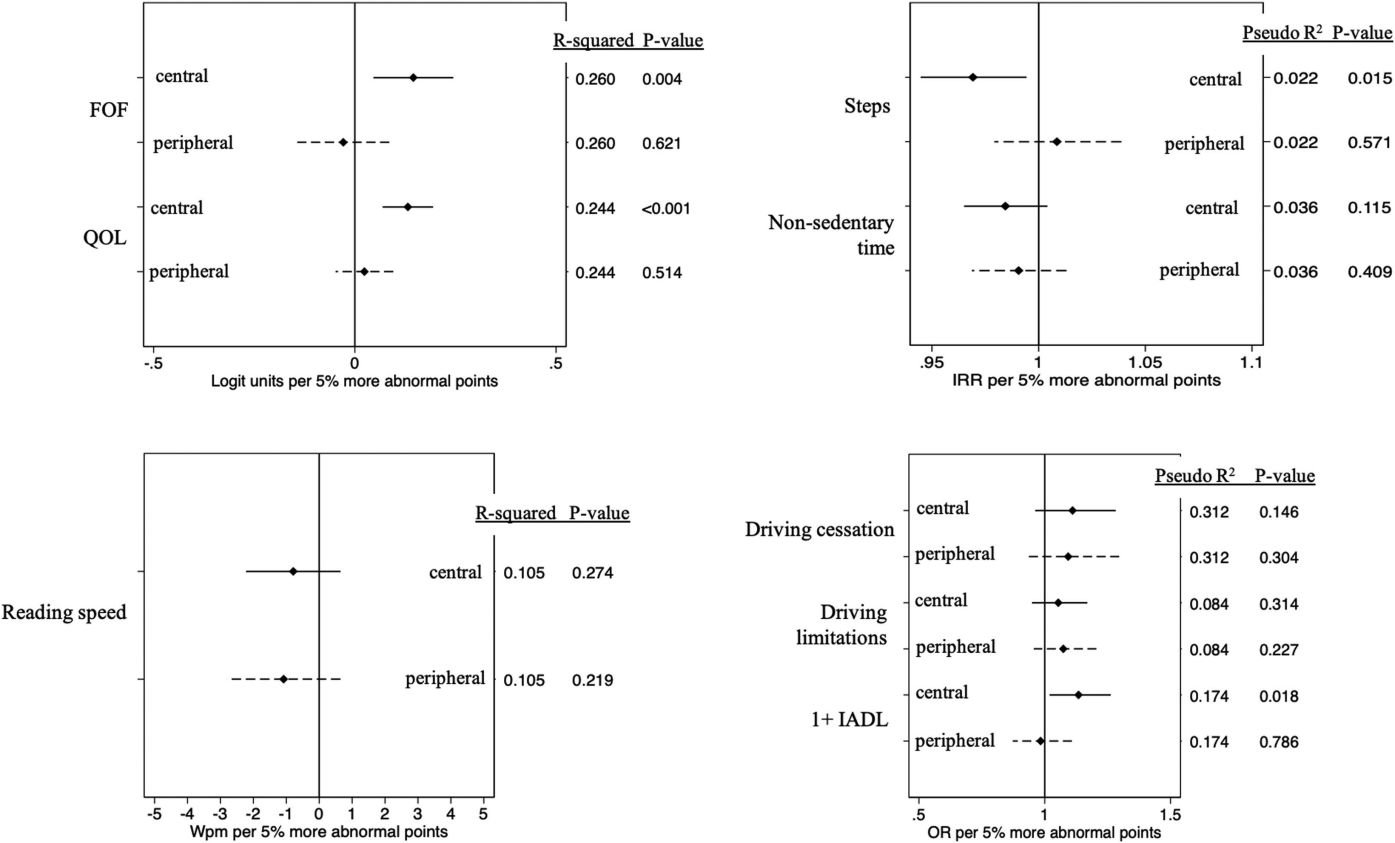
Independent effects of 5% more abnormal central or peripheral points on each functional outcome.

Multivariable models with both central and peripheral abnormal points included as independent variables were then used to evaluate the effects of central or peripheral VF damage independent of damage in the other region. In these models, greater central, but not peripheral, VF damage remained associated with greater fear of falling, worse quality of life, fewer daily steps, and difficulty with independent activities of daily living (Fig. 3 and Table 3; *P* < 0.02 for central; *P* > 0.5 for peripheral). In these modes, neither central nor peripheral damage was associated with maximum reading speed (*P* > 0.2 for each), less time in physical activity (*P* > 0.1 for each), driving cessation (*P* > 0.14 for each), or self-reported driving limitations (*P* > 0.2 for each).

**Table 3.** Evaluation of Disability With Central and Peripheral Damage as Independent Variables: Summary of Statistically Associated Functional Outcomes

Outcome Measure	Both	Central Only	Peripheral Only	Neither
FoF		X		
QOL		X		
Reading speed				X
Steps		X		
Nonsedentary time				X
Driving cessation				X
Driving limitations				X
1+ IADL		X		
Base of support				X
Step length			X	
Stride length			X	
Stride velocity				X
Walking speed				X
CV step length			X	
CV stride length				X
CV stride velocity				X

**Figure 4.**
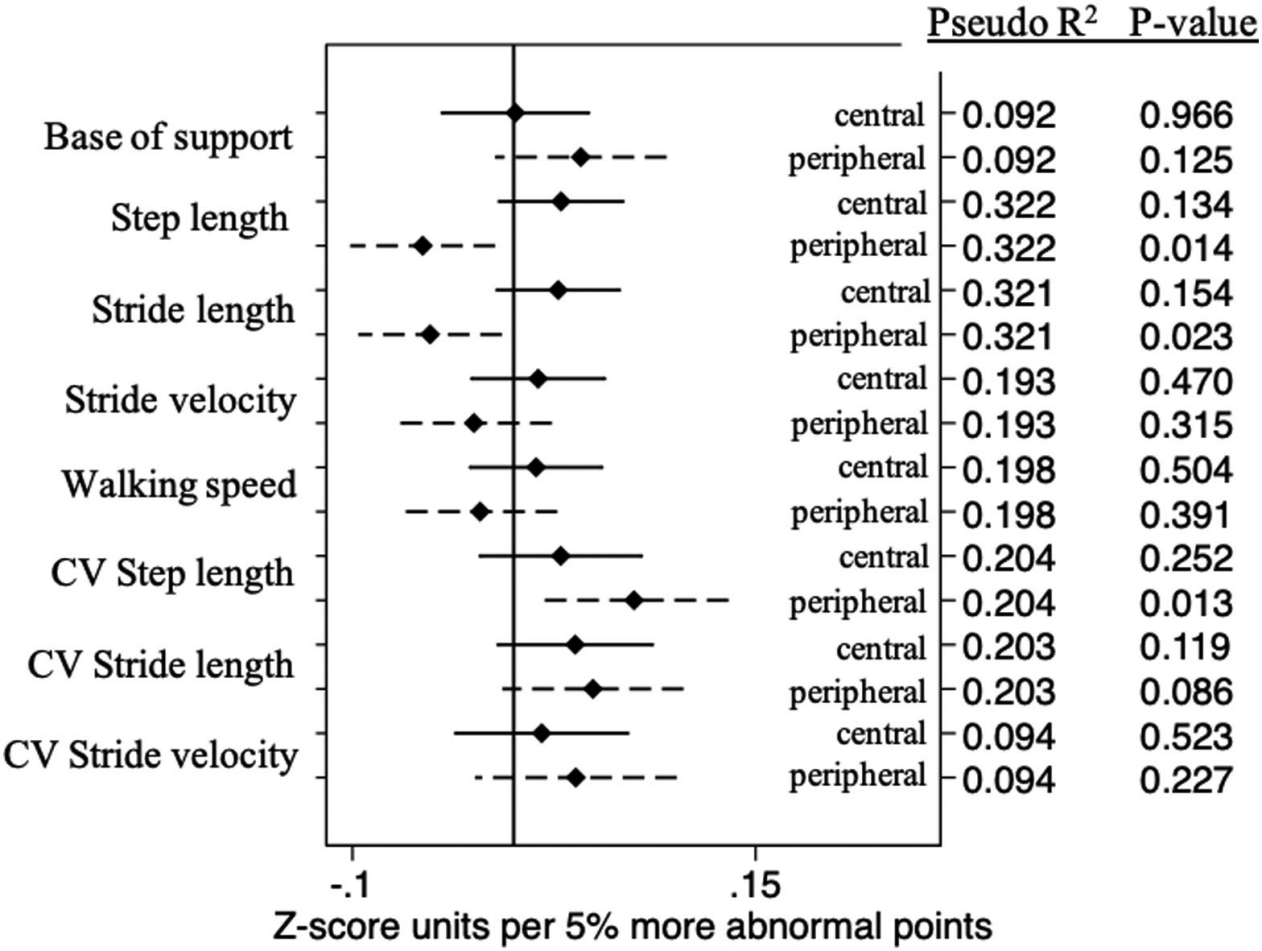
Independent effects of 5% more abnormal central or peripheral points on each gait outcome.

For gait measures, greater peripheral, but not central, damage remained associated with shorter steps and shorter strides and greater variability in step length (Fig. 4 and Table 3; *P* < 0.03 for peripheral; *P* > 0.13 for central). Neither central nor peripheral damage was associated with greater variability in stride length nor greater variability in stride velocity (Fig. 4 and Table 3; *P* > 0.08 for all).

For most outcomes assessed using linear regression, model *R*^2^ values were not substantially higher (less than 5% additional explained variability) for models including both central and peripheral VF damage as compared to the best assessing either central or peripheral damage.

### Sensitivity Analysis Using Standardized Values for Peripheral and Central Damage

As information is lost by dichotomizing central threshold, we ran further sensitivity analyses in which differences in disability measures were analyzed against the degree of central and peripheral VF damage in each test. Models were run to analyze differences in disability corresponding to a 5-dB decrement in central IVF sensitivity or 15 more abnormal points in the peripheral IVF (each corresponding to 1.15 *z*-score units).

The sensitivity analysis confirmed our main findings, with some exceptions. In the analyses including either central or peripheral damage, findings were the same for all outcomes except base of support; both peripheral and central damage were associated with greater base of support (*P* < 0.03), as opposed to our main findings in which only peripheral damage was associated (*P* = 0.02).

In analyses including both central and peripheral damage, some measures remained associated with central damage alone, while others remained associated with peripheral damage alone, although the list of these measures was slightly different.

Model *R*^2^ values from sensitivity analyses were not substantially different for the models that used the dichotomized central threshold values (main analysis).

## Discussion

In patients with glaucoma, the relative importance of central versus far peripheral VF damage to function differs across functional domains. As judged by the independent effects of peripheral or central VF damage on disability, central damage is more strongly associated with most disability outcomes (FoF, QOL, daily steps, and difficulty with IADLs), although peripheral VF damage is more strongly associated with specific gait characteristics such as step length, stride length, and greater variability in step length slightly better. For most outcomes, the differences between central versus peripheral VF damage (when considered as the only visual variable) are generally small, with only minor differences in *R*^2^ values. These findings suggest that it is difficult to disentangle the importance of more central and more peripheral VF damage and also that general blanket statements about which VF region (central or the more peripheral 30 to 60 degrees) is more relevant to disability should be avoided. Instead, a more nuanced approach is needed when considering the functional importance of the location of VF damage in which each functional domain is considered separately, and integrated measures of quality of life/activity limitation are eschewed in the absence of data, suggesting a consistent relationship between location of disability across domains.

Both central and peripheral vision loss, when analyzed in models in which only central or peripheral VF damage is considered, capture disability for several measures, including FoF, QOL, reading speed, physical activity level, driving cessation/limitations, and difficulty with IADLs. While there is no clear threshold for deciding when one measure (i.e., central versus peripheral VF damage) is more strongly associated with a given functional domain (e.g., fear of falling), one method is to compare the amount of explained variability (*R*^2^ values) across models. *R*^2^ values were consistently similar (less than 5% different) when models of disability based on central versus peripheral VF damage were compared. These findings likely reflect the moderate correlations between central and far peripheral VF damage, which allow for damage in any region to capture functional difficulties regardless of how directly they relate to the mechanism of disability, and highlight the challenges in making firm conclusions about which VF regions are most responsible for disability.

The relative importance of central and peripheral VF damage to disability can also be investigated by asking whether each affect disability independent of damage in the other region. Such analyses are potentially hampered by issues of collinearity, although VIFs in our statistical models were consistently less than 3. Of note, models accounting for the severity of both central and peripheral damage did not account for variability in disability measures to a meaningfully higher degree as compared to models including severity of VF damage in one location. Thus, while both peripheral and central regions are important in assessing function, neither one contributes a statistically significant amount of additional value. This reflects our prior point: by accounting for one region (whether far periphery or central 30 degrees), one to some extent captures all regions of VF loss, possibly since regions of VF loss are correlated, with additional visual measures not adding meaningful value.

Prior studies have shown that VF damage in the far periphery is moderately correlated with damage in the central 24 to 30 degrees (a variety of methods were used depending on the study: monocular and binocular, static and kinetic).^2^^,^^3^^,^^23^ However, only a few studies assess the impact of peripheral vision loss beyond the central 30 degrees on functional disability. Freeman et al. demonstrated that peripheral loss beyond the central 30 degrees was associated with falls, whereas loss within the central 30 degrees was not. While it may not be practical to measure peripheral vision clinically, it is not perfectly correlated with more central VF damage and, therefore, may add additional information in assessing specific functional outcomes, such as falls, gait, or other measures not studied here (i.e., motor vehicle accidents, pedestrian street crossing). One line of research might be to see if more peripheral damage could be judged using a limited number of peripheral points. While seemingly obvious, it should be kept in mind that all portions of the VF are important, although what they are important for may differ.

Our study adds to the paucity of literature available regarding the value of far peripheral damage in assessing functional disabilities by evaluating multiple metrics of disability, including objective ones (e.g., stride length, physical activity level) in a patient population with varying spectrum of glaucomatous disease. Most prior studies related vision measures to disability using a questionnaire assessing function across multiple functional domains.^8^^–^^10^ However, it should be kept in mind that the various activities queried (or directly measured in functional batteries such as those developed by George Spaeth)^24^ may not each have the same relationship to visual measures, calling into question the validity of judging the relationship between VF damage to summary measures of disability. For example, stronger associations of central VF damage with quality of life may be found if more tasks of central vision and fewer tasks of peripheral vision are included in the QOL questionnaire.

Study weaknesses include the challenge of comparing peripheral suprathreshold and central threshold testing.^2^ There was no perfect way of transforming peripheral VF data for comparison to central VF sensitivities as, due to the long length of peripheral threshold tests, we used a suprathreshold peripheral screening test that adjusts for the patients’ expected hill of vision, making it difficult to directly compare central and peripheral severity. By using pattern deviation values rather than total deviation values (as outlined in the Methods), we were best able to compare the importance of *localized* loss in the central and peripheral visual fields, although this approach would not be ideal in most other circumstances, as it obscures the impact of generalized loss, which also has an important impact on functionality. Of note, our sensitivity analysis that considered central VF damage via mean deviation (MD), as opposed to the number of abnormal points, showed very similar results. Additionally, we could have considered the location in each type of VF, since it has been shown that specific hemifields are more important for specific daily activities. For example, superior central vision is more important for driving abilities, whereas inferior central vision is more important for falls.^25^^–^^28^ Also, the inferior 10 to 30 degrees was more associated with quality of life^6^ and FoF^7^ compared to other areas of the central 30 degrees. However, we would have encountered collinearity issues if the VF was divided into too many subregions, as we have found that these models yield high VIFs (>3) in a study that used the same study population to look at the central VF only (unpublished results). Finally, we should be cautious about generalizing this study's findings to those with severe glaucomatous damage, since most patients had mild disease, although the spectrum ranged from early to late stages (Table 1), as previously described.^2^

Generally speaking, there are three reasons for VF testing—screening for disease, determining progression, and assessing function. Clinically, we currently focus on the first two more than the third, and practically, we want to tailor the type of VF to our clinical goals. However, preserving daily function has been a long-standing goal in glaucoma care, although more work is needed to judge how VF assessment can capture these goals.^1^ Further studies are also needed to address the question of whether certain functional deficits are related to more peripheral vision, which could help derive better estimates of a patient's disability level, which could inform clinicians about the level and nature of a patient's disability. If true, future central VF algorithms could obtain a limited amount of information regarding VF sensitivity in the far periphery, capturing this information while minimizing additional testing time and patient fatigue. Additionally, simulated environments may aid in capturing visual disability measures that are difficult to capture in multipronged questionnaires, such as feelings of fear and anxiety when a person is walking in poor lighting conditions.^29^^–^^31^

## Conclusion

The importance of central versus far peripheral VF damage in assessing function differs across functional domains in patients with glaucoma. Central damage was more strongly associated with most disability outcomes in this study, even for domains thought to be more dependent on peripheral vision (such as fear of falling). However, peripheral vision was slightly better for specific gait characteristics. For many of the variables, both central and peripheral visual field defects affected the outcome. Studies relating VF damage in specific regions to disability should separately consider disability in different domains as opposed to evaluating summary measures of disability.
